# Gender inequality and burden of orofacial clefts in the Eastern Mediterranean region: findings from global burden of disease study 1990–2019

**DOI:** 10.1186/s12887-024-04569-6

**Published:** 2024-01-23

**Authors:** Sara Sadat Nabavizadeh, Jennifer J. Mootz, Nasser Nadjmi, Benjamin B. Massenburg, Kaveh Khoshnood, Ehsan Shojaeefard, Hossein Molavi Vardanjani

**Affiliations:** 1 MD-MPH Department, School of Medicine, Shiraz University of Medical Sciences, Shiraz, Iran; 2 Otolaryngology Research Center, Shiraz University of Medical Sciences, Shiraz, Iran; 3 Department of Psychiatry, Columbia University, 1051 Riverside Drive, New York, NY 10032 USA; 4 New York State Psychiatric Institute, 1051 Riverside Drive, Kolb 171, New York, NY 10032 USA; 5 Department of Cranio-Maxillofacial Surgery, Antwerp University Hospital, Antwerp, Belgium; 6 Department of Maxillofacial Surgery, ZMACK, AZ MONICA Antwerp, Antwerp, Belgium; 7 Department of Surgery, Division of Plastic and Reconstructive Surgery, University of Washington, Seattle, WA USA; 8 School of Public Health, Yale University, 60 College St, New Haven, CT 06510 USA; 9 Research Center for Traditional Medicine and History of Medicine, School of Medicine, Shiraz University of Medical Sciences, Shiraz, Iran

**Keywords:** Prevalence, Gender inequality, Orofacial clefts, Pediatrics, Disability-adjusted life years

## Abstract

**Background:**

Gender inequality may be associated with the burden of orofacial clefts (OFCs), particularly in low-and middle-income countries (LMICs). To investigate the OFCs’ burden and its association with gender inequality in the Eastern Mediterranean region (EMR).

**Methods:**

Country-specific data on the OFCs’ prevalence and Disability-Adjusted Life Years (DALYs) from 1990 to 2019 were gathered from the Global Burden of Disease database by age and gender. Estimated annual percentage change (EAPCs) was used to investigate the OFCs’ trends. The association of the Gender Inequality Index (GII) with prevalence and DALY rates was determined using multiple linear regression. Human Development Index (HDI), Socio-Demographic Index (SDI), and Gross Domestic Product (GDP) were also considered as potential confounders.

**Results:**

In 2019, the overall regional OFCs’ prevalence and DALYs (per 100,000 person-years) were 93.84 and 9.68, respectively. During the 1990–2019 period, there was a decrease in prevalence (EAPC = -0.05%), demonstrating a consistent trend across genders. Moreover, within the same timeframe, DALYs also declined (EAPC = -2.10%), with a more pronounced reduction observed among females. Gender differences were observed in age-specific prevalence rates (p-value = 0.015). GII was associated with DALYs (β_male_= -0.42, p-value = 0.1; β_female_ = 0.48, p-value = 0.036) and prevalence (β_male_= -1.86, p-value < 0.001, β_female_= -2.07, p-value < 0.001).

**Conclusions:**

Despite a declining prevalence, the burden of OFCs remained notably significant in the EMR. Gender inequality is associated with the burden of OFCs in the Eastern Mediterranean region. Countries in the region should establish comprehensive public policies to mitigate gender inequalities in healthcare services available for OFCs.

**Supplementary Information:**

The online version contains supplementary material available at 10.1186/s12887-024-04569-6.

## Introduction

Orofacial clefts (OFCs) including cleft lip and palate are among the most common congenital head and neck anomalies, accounting for 652,000 disability-adjusted life years (DALYs) worldwide [1]. In addition to debilitating physical effects, such as appearance, speech, and hearing, OFCs can expose children to several psychological problems like anxiety and depression. Moreover, the stigma attached to OFCs disadvantages afflicted children in education, employment, marriage, and community [2]. Addressing the needs of children with OFCs necessitates a multidisciplinary approach due to their susceptibility to diverse health and mental health adversities. However, providing comprehensive care poses financial challenges for both healthcare systems and patients’ families, particularly in resource-constrained settings [3].

The disproportionate burden of OFCs is prevalent in low- and middle-income countries (LMICs), accounting for about 84.0% of cases and nearly 94.1% of the overall disease burden attributed to OFCs [1].

Financial constraint is the primary impediment to comprehensive cleft care delivery in LMICs [4]. Notably, financial resources are unequally distributed across genders, resulting in gender discrepancies in the utilization of screening services and other facilities. Gender inequalities affect health outcomes from early life due to differential healthcare access, health-risk behaviors, gender biases in healthcare systems, and unequal resource allocation in health research and data collection [5–8]. Accordingly, gender discrepancies may lead to a higher burden attributed to OFCs among female patients [9].

Most countries in the Eastern Mediterranean Region (EMR) are categorized as low- and middle-income countries. Although these countries differ in terms of gross domestic product (GDP), socioeconomic standing, and degree of gender disparity, they all share nearly the same genetic foundation, culture, and behavioral tendencies and are categorized as in-transition countries. Previous research has suggested that EMR countries face higher levels of gender inequality than countries in most other parts of the world [10]. In EMR countries, women have lower levels of education, employment, health literacy, health facility utilization, and poorer health outcomes than men [11].

Many barriers are associated with providing efficient and effective care for patients with OFCs in the EMR. Identifying factors associated with the OFC’s burden in the EMR would help regional planners, implementers, and assistance organizations in taking evidence-informed and targeted action for vulnerable populations. This study aimed to investigate the correlation of gender inequality with the burden of OFCs in the EMR. Additionally, we sought to assess changes in the prevalence and DALYs of OFCs in the EMR over the preceding three decades based on age groups and gender, compared with the global trends.

## Materials and methods

We analyzed the most updated ecological data available on the burden of OFCs and gender inequality in the EMR between 1990 and 2019.

The Global Health Data Exchange (GHDx) query tool was used to find information on the disease burden of OFCs (available at: http://ghdx.healthdata.org/gbd-results-tool). Data from the Global Burden of Diseases (GBD) project, conducted by the Institute for Health Metrics and Evaluation (IHME), are included in this online tool. GBD is an ongoing global collaboration that utilizes all available epidemiological data to provide a comparative assessment of health loss caused by 328 diseases in 195 countries and territories. The GBD methodology is detailed on its official website (http://www.healthdata.org/gbd).

In brief, the GBD group utilized an optimized Bayesian meta-regression model to estimate the prevalence of OFCs, encompassing isolated cleft lip, isolated cleft palate, and combined cleft lip and palate as distinct subgroups. These estimations served as inputs for an incidence-prevalence-mortality model within the GBD-2017 study framework [12].

Furthermore, the calculation of DALYs for OFCs involved assigning disability weights to different conditions, such as unrepaired symptomatic clefts causing stress, speech issues linked to unrepaired clefts or their consequences, partially repaired clefts leading to long-term sequelae, and completely repaired asymptomatic clefts without sequelae. To capture the uncertainty of these estimations, a repeated sampling approach was employed, generating years lived with disability computed through 1000 iterations, each iteration drawing a unique sample from the data.

[1] Data on the prevalence and DALYs were obtained for 22 countries located in the EMR. Age and gender-specific rates, as well as age-standardized rates (ASR), and their uncertainty intervals (UI), were retrieved. Age groups were defined as less than one year, 1–4 years, 5–9 years, 10–14 years, 15–19 years, 20–24 years, and older than 25 years. Worldwide data was also retrieved to compare the patterns in the EMR with the global patterns.

Estimated Annual Percentage Change (EAPC) was utilized to quantify the trends of the age-standardized prevalence and DALYs of OFCs. To estimate the EAPC, a regression line was fitted to the natural logarithm of rates: *ln (rate) = a + βx + e*, where *x* is the calendar year, y = ln (rate), *e* depicts error, and *a* is the intercept. EAPC is then calculated as 100 × (*exp (β)*-1), and its 95% confidence interval (CI) is also obtained from a linear regression model [13].

We used the United Nations Development Program’s Gender Inequality Index (GII; GII-2019) to quantify gender inequality. It quantifies gender disparities in three domains: reproductive health, women’s empowerment, and economic status. This indicator runs on a continuous scale ranging from 0 to 1, with 0 signifying gender equality and 1 indicating total inequality for a particular gender across all measured variables [14].

The Human Development Index (HDI), a composite index of social and economic achievement, was also obtained at the national level in 2019 using the United Nations Development Program’s database. The HDI comprises four components: a life expectancy index, a mean number of years spent in education, the expected number of years spent in school, and an income index. The Socio-Demographic Index (SDI) measures a country’s development status based on education, fertility, and poverty levels and was obtained from the GHDx’s 2019 open database (http://ghdx.healthdata.og/). Gross domestic product (GDP) per capita was retrieved using purchasing power parity (constant 2011 international $) from the World Bank’s open database (http://data.worldbank.org/).

The average of delta SDI, a potential confounder, and a proxy measure of the variation of countries’ human development was computed as the value of the SDI in 2019 minus the SDI in the first year for which the SDI value was available, divided by the number of years of the calculated period, and included into the multivariable models. This variable was considered to be an indicator of the intensity and magnitude of a country’s development progression. We hypothesized that the associations between GII and the OFC disease burden in countries with the same development index value may be confounded or moderated by the extent and severity of the country’s progress toward human development during the last decades.

### Statistical analysis

The Wilcoxon signed-rank test was used to compare the rates. Crude measure of association between GII and the DALYs/prevalence of OFCs was assessed using ordinary least square (OLS) regression and demonstrated by scatter plots. Data was stored in a long hierarchical format. This format provided us an opportunity to consider the interrelationships of the data available for each country, which means that reported standard errors were estimated by applying the variance-covariance matrix of the estimators (VCE) which allows for intragroup correlation.

To elucidate the adjusted association between the GII and the DALYs as well as prevalence rates related to OFCs within the EMR, multivariable regression models were employed. Our analysis incorporated DALY and prevalence rates spanning the years 1990 to 2019, capturing the longitudinal trends across this time frame. Based on the available data, potential confounders/moderators including the 2019 SDI, changes in SDI (ΔSDI), 2019 HDI, changes in HDI (ΔHDI), 2019 GII, interaction term of HDI and GII, 2019 GDP per capita, changes in GDP (ΔGDP), and year were included in the multivariable models as independent factors. Through a backward elimination process, the 2019 SDI, changes in HDI (ΔHDI), 2019 GDP per capita, and changes in GDP (ΔGDP) variables were excluded based on P-value greater than 0.1. The final regression models were constructed with independent variables including the 2019 HDI, 2019 GII, change in SDI (ΔSDI), interaction term of HDI and GII, and year. Specifically, six OLS models were developed to explore the individual relationship between GII and the prevalence/DALYs, stratified by gender categories (male, female, and combined), to comprehend the nuanced impact of gender inequality on the burden of OFCs within the EMR. Multicollinearity was assessed using a variance inflation factor (VIF) greater than 1.5.

In addition to assessing the HDI interaction with other country-level variables, we repeated regression modeling in three strata including countries with high, medium, and low HDI scores. A p-value less than 0.05 was considered statistically significant. The analysis was conducted using the Python software (version 3.8.7 by the Python Core Team).

## Results

### Prevalence

**Fig. 1:**
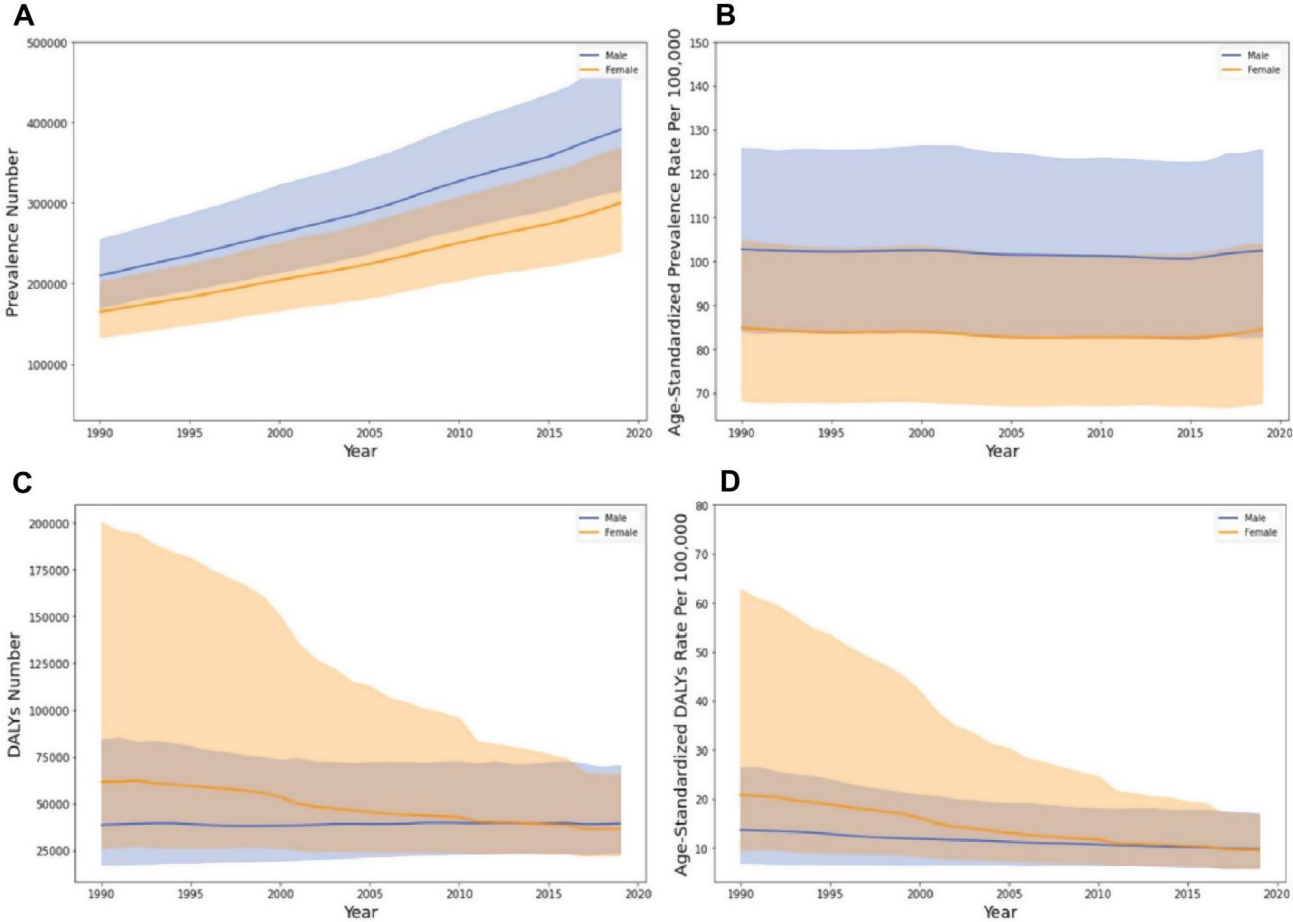
The time trend of orofacial clefts prevalence (**A**, **B**) and disability-adjusted life years (**C**, **D**) in the Eastern Mediterranean region from 1990 to 2019

Despite a substantial increase in the absolute prevalence of OFCs over the last three decades, reaching an estimated 691 × 10^3^ (UI: 560 to 851 × 10^3^) cases with a rate of 95.29 (UI: 77.19 to 117.23 per 100,000) in 2019, there was a marginal decrease in the age-standardized prevalence rate observed between 1990 and 2019 (EAPC= -0.05%, CI: -0.06% to -0.05%; Table 1). Notably, both males and females exhibited a similar decreasing trend in age-standardized prevalence rates independently (male EAPC= -0.05%, CI: -0.06% to -0.04%; female EAPC= -0.06%, CI: -0.07% to -0.05%), although males consistently demonstrated a higher prevalence age-standardized rate compared to females across all years (Fig. 1A and B, Fig. S1A). However, given the EAPC approaching zero, it is apparent that there has been minimal progress in reducing the incidence of clefts at birth within the EMR.

**Table 1:** Orofacial clefts prevalence and disability-adjusted life years in 1990 and 2019, with estimated annual percentage change

Location	Measure	Sex	Number 1990 × 10^5^ (UI)	ASR 1990 (UI) Per 10^5^	Number 2019 × 10^5^ (UI)	ASR 2019 (UI) Per 10^5^	EAPC (95% CI)	P-value
Global	Prevalence	Male	17.1 (14.0 to 20.9)	61.37 (50.01 to 74.82)	24.1 (19.7 to 29.6)	62.77 (51.28 to 76.92)	0.11 (0.10 to 0.12)	< 0.001
		Female	15.6 (12.7 to 19.0)	56.94 (46.44 to 69.54)	22.1 (17.9 to 27.2)	58.39 (47.59 to 71.62)	0.17 (0.16 to 0.19)	< 0.001
		Both	32.7 (26.8 to 39.9)	59.14 (48.28 to 72.15)	46.2 (37.6 to 56.7)	60.60 (49.51 to 74.45)	0.14 (0.13 to 0.16)	< 0.001
	DALYs	Male	6.87 (3.33 to 11.7)	21.04 (10.45 to 35.28)	2.73 (1.82 to 4.65)	7.58 (5.03 to 13.44)	-3.73 (-3.78 to -3.67)	< 0.001
		Female	5.59 (3.69 to 11.1)	18.14 (12.12 to 35.68)	2.57 (1.68 to 4.25)	7.45 (4.84 to 12.73)	-3.10 (-3.16 to -3.04)	< 0.001
		Both	12.5 (8.07 to 17.5)	19.63 (12.85 to 27.44)	5.30 (3.62 to 7.98)	7.51 (5.10 to 11.57)	-3.44 (-3.50 to -3.38)	< 0.001
Eastern Mediterranean Region	Prevalence	Male	2.10 (1.70 to 2.55)	102.75 (84.05 to 125.91)	3.91 (3.16 to 4.79)	102.41 (82.88 to 125.62)	-0.05 (-0.06 to -0.04)	< 0.001
		Female	1.65 (1.34 to 2.02)	84.86 (68.42 to 104.79)	3.00 (2.40 to 3.68)	84.56 (67.66 to 104.01)	-0.06 (-0.07 to -0.05)	< 0.001
		Both	3.75 (3.06 to 4.56)	94.14 (76.40 to 114.81)	6.91 (5.60 to 8.51)	93.84 (75.98 to 115.49)	-0.05 (-0.06 to -0.05)	< 0.001
	DALYs	Male	0.386 (0.173 to 0.843)	13.62(6.86 to 26.46)	0.394 (0.239 to 0.705)	9.77 (5.98 to 17.06)	-1.16 (-1.18 to -1.15)	< 0.001
		Female	0.615 (0.263 to 2.00)	20.80 (9.61 to 62.77)	0.366 (0.224 to 0.657)	9.58 (5.90 to 16.72)	-2.90 (-2.95 to -2.86)	< 0.001
		Both	1.00 (0.484 to 2.27)	17.12 (8.99 to 36.13)	0.760 (0.486 to 1.24)	9.68 (6.23 to 15.47)	-2.10 (-2.13 to -2.07)	< 0.001

*Abbreviations: EAPC: Estimated Annual Percentage Change; DALYs: Disability-adjusted life years; ASR: Age-standardized rate; 95%CI: 95% confidence interval; UI: uncertainty interval

**Fig. 2:**
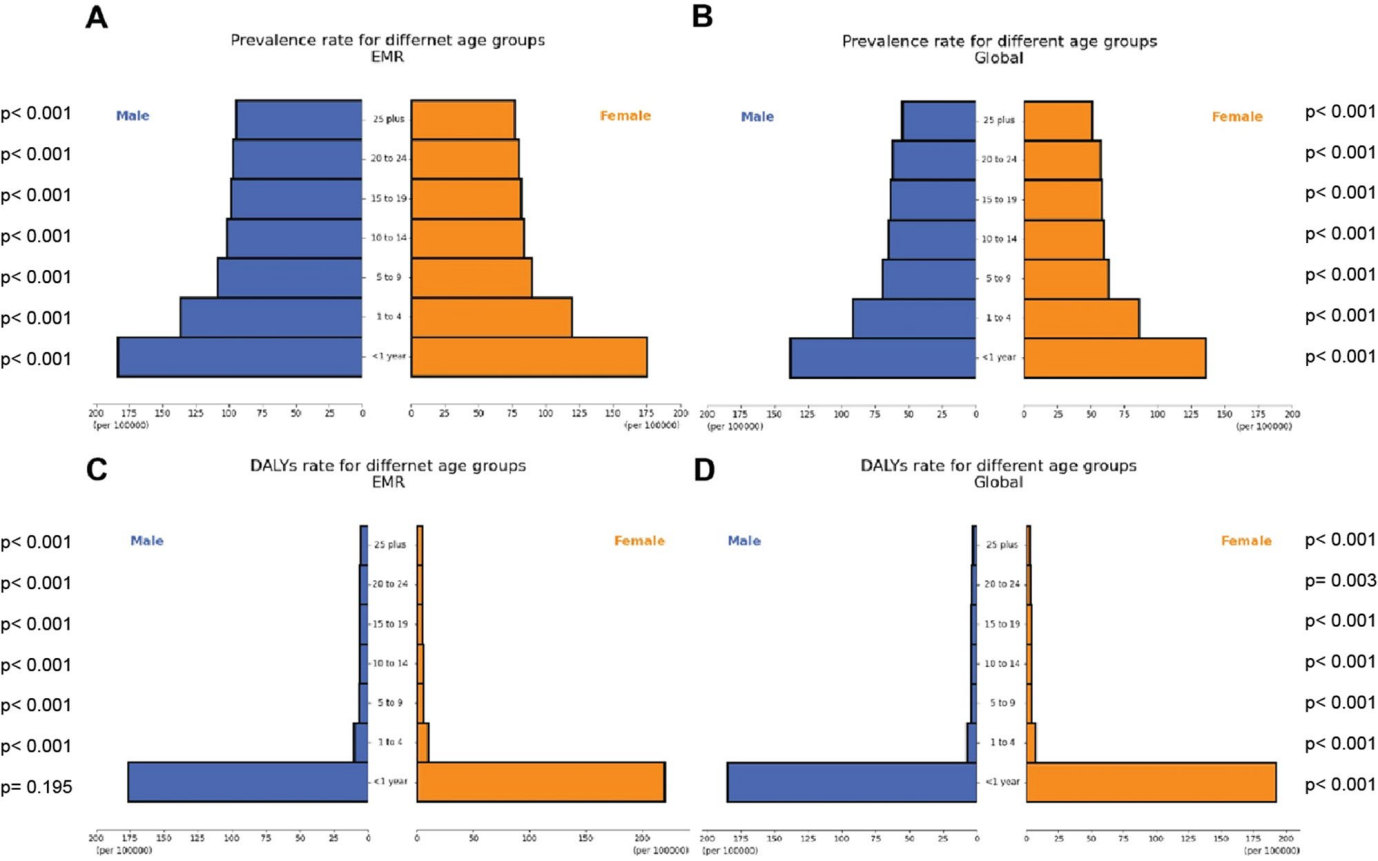
Orofacial clefts’ prevalence (**A**, **B**) and disability-adjusted life years (**C**, **D**) in specified age groups in the Eastern Mediterranean region-2019 (EMR)

Analysis of prevalence rates for both genders in various age groups revealed that in each age group, the prevalence rate was considerably greater for males than for females (p-value = 0.015; Fig. 2A and B).

In contrast to the EMR trend, the worldwide prevalence of age-standardized rate has slightly grown during the past thirty years and reached 60.60 (UI: 49.51 to 74.45) cases per 100,000 population in 2019. Moreover, after comparing EMR with the world in the context of age group, in all age groups, the prevalence rate was higher in EMR than in global (Fig. 2A and B).

### Disability-adjusted life-years

Between 1990 and 2019, the DALYs cases and age-standardized rate decreased (EAPC= -2.10%, CI: -2.13% to -2.07%), reaching 76 × 10^3^ (UI: 46 to 124 × 10^3^; rate: 10.46, UI: 6.70 to 10.46 per 100,000) and 9.68 (UI: 6.23 to 15.47 per 100,000) respectively in 2019 (Table 1). Although both males and females exhibited a decrease in DALYs age-standardized rate, females experienced a more pronounced drop (male EAPC= -1.16%, CI: -1.18% to -1.15%, female EAPC= -2.90%, CI: -2.95% to -2.86%; Fig. 1C and D, Fig S1B).

Analysis of DALY rates for both genders in various age groups revealed no differences (p-value = 0.296; Fig. 2C and D).

The global DALYs age-standardized rate was higher in 1990 than in the EMR, while this ratio was reversed in 2019 due to the steeper slope of the global trend (Global EAPC= -3.44%, CI: -3.50% to -3.38% vs. EMR EAPC= -2.10%, CI: -2.13% to -2.07%). Furthermore, after comparing EMR with the world in the context of age group, no significant difference was observed except for less than 1 y/o, which was minimally higher global than EMR (Fig. 2C and D).

### The burden of the orofacial cleft in EMR, national level 1990–2019

Online Supplementary Table 1 lists the prevalence and DALYs and their age-standardized rate of OFCs in 22 EMR countries in 1990 and 2019 (Table S1).

In 2019, among the EMR’s 22 nations, Pakistan had the most significant absolute prevalence number of orofacial clefts, and Djibouti had the lowest (2.38 × 10^5^, UI: 1.91 × 10^5^ to 2.94 × 10^5^ (rate: 106.38, UI: 85.27 to 131.42 per 100,000),5.77 × 10^2^, UI: 4.62 × 10^2^ to 7.20 × 10^2^ (rate: 47.97, UI: 38.44 to 59.82 per 100,000); respectively).

The prevalence age-standardized rate varied slightly throughout the EMR, with Palestine having the greatest prevalence and Djibouti having the lowest (139.41; UI: 113.56 to 171.37, 46.59; UI: 37.15 to 58.26 per 100,000; respectively).

**Fig. 3:**
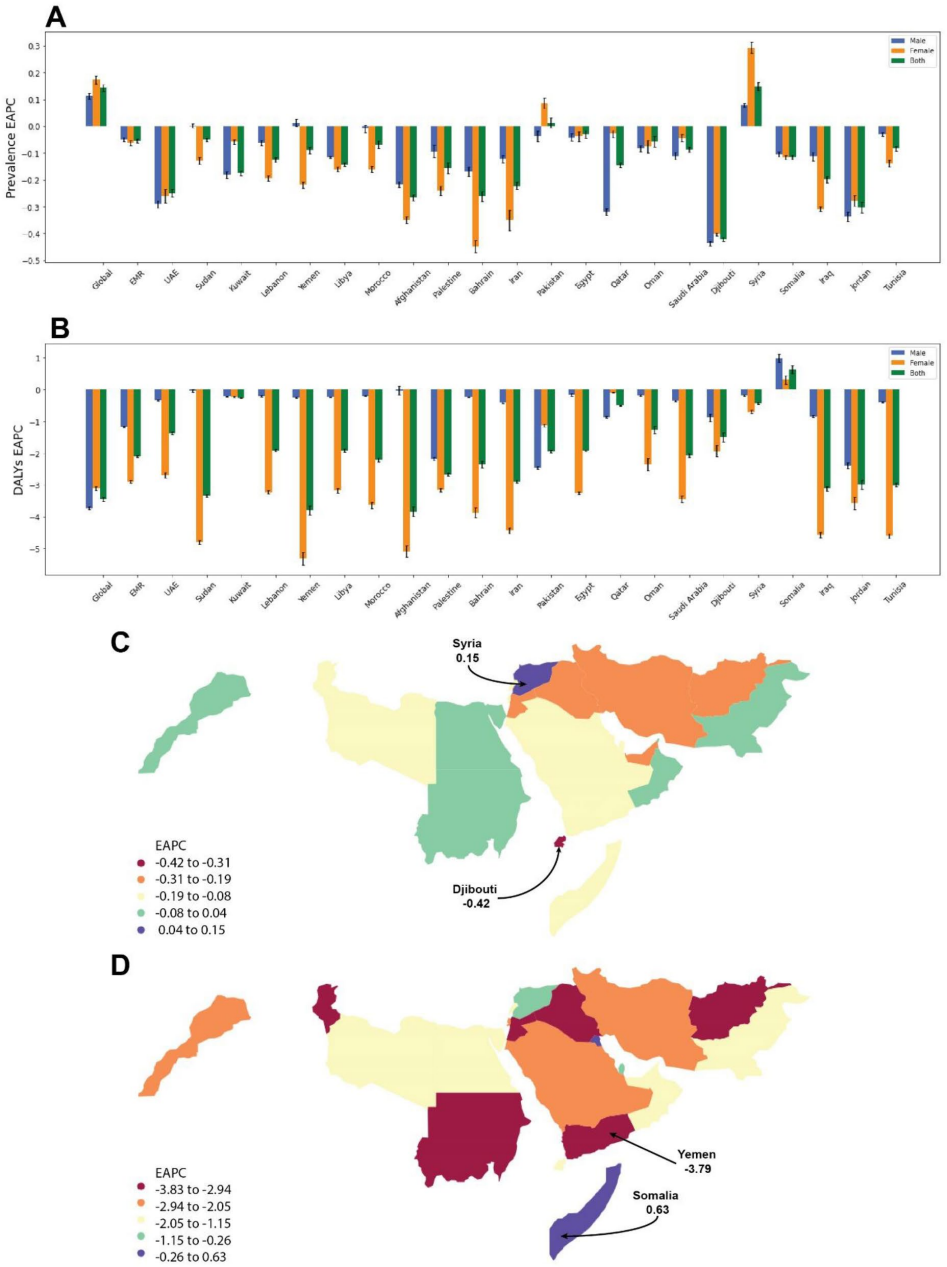
Estimated annual percentage change in prevalence (**A**) and disability-adjusted life years (**B**) for the Eastern Mediterranean region, the world, and all 22 Eastern Mediterranean region countries. World map of estimated annual percentage changes (EAPC) of orofacial clefts prevalence (**C**) and disability-adjusted life years (**D**), 1990–2019. Countries with extreme values were annotated

In most EMR countries, the age-standardized prevalence has remained relatively steady throughout time, with only minor increases or decreases. Syria demonstrated the most pronounced upward trend (EAPC = 0.15%, CI: 0.13–0.16%), while Djibouti had the most significant downward trend (EAPC= -0.42%, CI: -0.43% to -0.41%). Although the prevalence rate was higher in males than in females in all nations in 1990 and 2019, gender variations in the prevalence trend slope were negligible in almost all countries (Fig. 3A and C).

In 2019, among the EMR’s 22 nations, Pakistan had the most significant absolute DALYs number, while Bahrain had the lowest (3.08 × 10^4^, UI: 1.69 × 10^4^ to 5.33 × 10^4^ (rate: 13.51, UI: 7.54 to 23.78 per 100,000), 8.73 × 10^1^, UI: 5.51 × 10^1^ to 1.30 × 10^2^ (rate: 6.04, UI: 3.82 to 8.98 per 100,000); respectively). The DALYs age-standardized rate varied markedly across the EMR countries, the highest in Somalia and the lowest in Iran (18.00, UI: 4.51 to 71.60, 5.74, UI: 3.8˜6 to 8.13 per 100,000; respectively).

Between 1990 and 2019, all countries demonstrated a declining trend in the DALYs age-standardized rate except for Somalia, while Yemen had the most dramatic decline (-61.36%; EAPC= -3.79%, CI: -3.94% to -3.64%) which was accounted for almost exclusively by females.

Additionally, the male-to-female ratio of DALYs was steady, with women having greater DALYs than males in most nations in 1990. However, this ratio was flipped in 2019, except for Pakistan (male: female = 0.83), Afghanistan (male: female = 1.70), Djibouti (male: female = 1.31), Sudan (male: female = 1.21), Yemen (male: female = 1.32), and Somalia (male: female = 1.05). There were also gender differences in the declining trend between 1990 and 2019, with females seeing a considerably greater rate of decline than males. However, in Qatar, Pakistan, and Somalia, the declining trend for males is steeper (Fig. 3B and D).

### The burden of the orofacial cleft and gender inequality

**Fig. 4:**
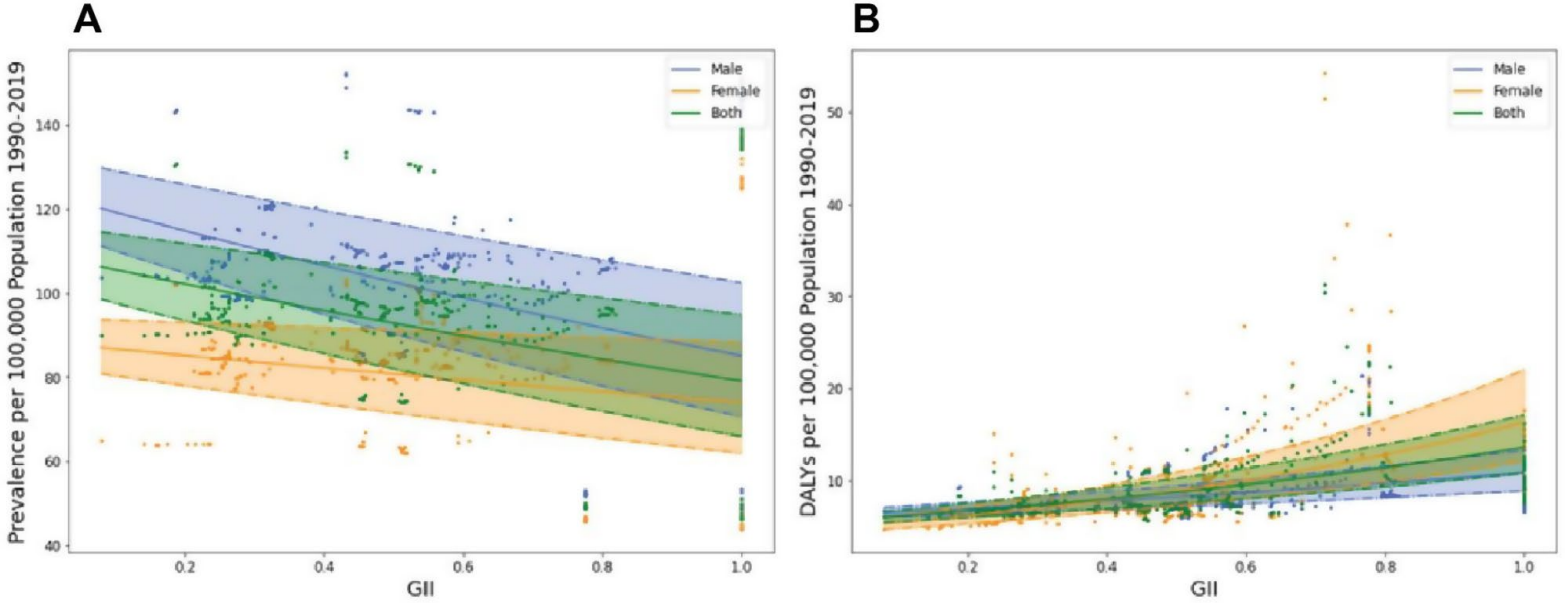
Correlation between gender inequality index and orofacial clefts 1990–2019 prevalence (**A**) and disability-adjusted life years (**B**) rate

The GII and the trend in DALYs over the last thirty years as an indicator of orofacial clefts disease burden revealed a moderate positive association (r^2^ = 0.334, p-value < 0.001). Conversely, the prevalence trend demonstrated a slight negative correlation with GII in EMR (r^2^ = 0.095, p-value < 0.001; Fig. 4).

Multivariable linear regression model demonstrated a positive correlation between GII and DALYs in females and both genders (female adjusted β = 0.48, p-value = 0.036, both adjusted β = 0.25, p-value = 0.245) while a negative correlation with prevalence (male adjusted β= -1.86, p-value < 0.001, female adjusted β= -2.07, p-value < 0.001, both adjusted β= -1.75, p-value < 0.001) and DALYs in males (adjusted β= -0.42, p-value = 0.1; Table 2; Fig. S2).

**Table 2:** Correlation between country-level indicators and orofacial clefts 1990–2019 prevalence and rate of disability-adjusted life years

			2019 GII	2019 HDI	Δ SDI	GII-HDI Interaction	Year
Male	DALYs	Crude β	0.41	-0.46	-0.54	0.09	-0.15
		Adjusted β	-0.42	-0.049	-0.53	0.39	-0.076
		Std Error	3.56	3.25	2.7	5.02	0.019
		P-value*	0.10	0.002	0.00	0.023	0.082
	Prevalence	Crude β	-0.25	0.539	0.55	0.19	-0.52
		Adjusted β	-1.86	-0.64	0.20	1.54	-0.07
		Std Error	23.71	21.64	17.93	33.38	0.13
		P-value*	< 0.001	< 0.001	< 0.001	< 0.001	0.09
Female	DALYs	Crude β	0.48	-0.68	-0.33	0.06	-0.42
		Adjusted β	0.48	-0.35	-0.1	-0.39	-0.311
		Std Error	6.69	6.1	5.06	9.42	0.03
		P-value*	0.036	0.027	0.034	0.00	0.00
	Prevalence	Crude β	-0.05	0.33	0.50	0.30	-0.07
		Adjusted β	-2.07	-0.98	0.24	1.78	-0.03
		Std Error	19.83	18.1	15	27.92	0.10
		P-value*	< 0.001	< 0.001	< 0.001	< 0.001	0.44
Both gender	DALYs	Crude β	0.50	-0.71	-0.46	0.08	-0.40
		Adjusted β	0.25	-0.37	-0.28	-0.18	-0.266
		Std Error	4.04	3.69	0.022	5.69	0.022
		P-value*	0.245	0.004	0.00	0.253	0.00
	Prevalence	Crude β	-0.20	0.50	0.54	0.22	-0.06
		Adjusted β	-1.75	-0.62	0.21	1.50	0.066
		Std Error	22.15	20.22	16.76	31.19	0.122
		P-value*	< 0.001	< 0.001	< 0.001	< 0.001	0.13

Abbreviation: DALYs: Disability-adjusted life years; GII: gender inequality index; HDI: human developmental index; SDI: socio-demographic index; Std Error: standard error

*Estimated for adjusted β

## Discussion

This study quantified the consequences of gender inequality on orofacial cleft population health indices in the EMR. In addition, we investigated the prevalence and DALYs trend from 1990 to 2019 in males, females, and both genders and compared it with the global trend. Our findings imply that the correlation of gender disparity with the burden and prevalence of orofacial clefts should be considered. In almost all models, gender equity was strongly associated with better outcomes for DALYs and prevalence for the whole population and males and females separately [14]. While regional variations in orofacial clefts and illness burden have been described earlier [1], our study integrates this information with accessible country-level indices and gender inequality data.

The study’s findings indicate that while orofacial prevalence has remained relatively stable over the previous three decades, the EMR’s DALYs showed less reduction than globally. The difference in DALYs decrease may be related to a lack of orofacial clefts screening, surgical and medical facilities, inadequate quantity and quality of health workforce, mistrust in the health care system, and gender inequality in low and middle-income countries [1, 11, 15]. In many low- and middle-income countries, medical record registration systems confront significant obstacles that jeopardize the reliability and quality of the data they supply [16]. Additionally, many cleft-related consequences, including dental decay, tooth loss, and hearing loss, have not been included in the orofacial clefts burden analysis, resulting in a conservative assessment of the disease’s worldwide burden. Furthermore, as a result of significant but differential rates of underestimation of DALYs in different LMICs, the disparity in DALYs between countries within different income clusters may be underestimated [1]. Therefore, to overcome the current challenges faced by countries in the EMR, long-term priorities should be to strengthen their healthcare workforce, establish multidisciplinary orofacial clefts centers that monitor patients from birth for screening, surgical palatoplasty, and subsequent follow-up, increase public trust in the health system, and reduce health-related gender inequality.

Our findings indicate that gender disparity at the national level has a negative correlation with the prevalence of orofacial clefts in males, females, and both genders, but a positive association with DALYs in the female population. The GII is driven by societal issues in which women confront disadvantages in terms of health, employment, and political influence [17]. To compensate for the influence of general socioeconomic factors, excluding those related to gender concerns, the HDI and other country-level metrics were first incorporated into the model. The influence of GII grows when HDI is added to the statistical prevalence regression model, as seen by the change in the standardized coefficients. Due to the significant association between HDI and GII, the findings indicate that gender inequality alone may be a macro-determinant of orofacial cleft prevalence and burden in EMR nations.

Economic studies document a U-shaped cross-country association between economic development and gender equality. This suggests that while economic progress will eventually increase gender equality, it will initially result in gender disparity as a consequence of decreased female labor force participation [18, 19]. By examining the trends of the OFCs prevalence over the last three decades and its relationship to gender inequality at all levels of gender, we hypothesized that some countries in EMR are at a level of social development that enables them to provide adequate diagnosis and screening services to both sexes equally. However, the attributed disease DALYs rate was correlated with gender inequality only in females, indicating that gender imbalance occurs in the timely diagnosis and post-diagnostic care of females.

Although the prevalence experienced a decreasing trend over time in both genders, the slope was steeper among females. Despite the higher prevalence rate in males compared with females, the DALYs rate in females is still higher. This result would magnify that gender inequality has a greater impact on females than males. There are several pathways in which gender inequality could affect the orofacial cleft burden. Epidemiological studies have suggested that while males and females have higher cleft-lip and cleft-palate ratios, respectively, parents of children born with cleft-lip were significantly more likely to seek care than parents of children born with isolated cleft-palate, owing to the palatal defect’s less visible nature, resulting in delayed diagnosis in females [20–22]. Additionally, in developing societies such as those in the EMR, parents are more inclined to seek medical treatment for a sick boy than a sick girl, owing to patrilocality and patrilineality concerns [18] Another study found that primary palatoplasty (primary lip, palatal, and alveolar repair) was equally prevalent in males and females, but secondary palatoplasty (aesthetic and functional revision), was considerably skewed toward females due to social stigma-induced beauty satisfaction [23] The issue of gender inequality in OFC is further complicated by that there are no epidemiological data available regarding the different subtypes of OFC, CLP, and CP. Counting CLP and CP in the single entity of OFC can confound the real issues of gender disparity among the various features of this disorder, from neonatal diagnosis, suitability, and type of surgery to prognosis. To date, no study has evaluated the epidemiological characteristics of different subtypes of OFC and the deficiency of data about this matter is a widespread limitation of these studies and also the GBD [1, 24] Therefore, collecting more epidemiological data on the OFC subtypes is highly needed to deal with these two major subgroups of OFC as separate entities. While existing literature highlights sex-based differences in the prevalence of OFCs, our decision to conduct separate analyses for males and females stems from the necessity to delve deeper into the nuanced aspects of gender disparities within this condition. Although general prevalence rates may demonstrate variations between genders, the underlying factors influencing diagnosis, treatment approaches, and long-term outcomes could potentially exhibit multifaceted distinctions between males and females. Moreover, while the prevalence of OFCs might differ between genders, the impact of sociocultural factors, access to healthcare, and individual responses to treatment remains understudied concerning sex-specific variations. Therefore, our decision to perform separate analyses is driven by the intent to explore beyond the surface-level prevalence differences and uncover potential gender-specific intricacies that may significantly influence the diagnosis, management, and prognosis of orofacial clefts.

A significant strength of this research is the robustness of the findings, as the analysis covered all EMR nations over an extended period. Thus, the findings represent a range of socio-historical circumstances. Moreover, all data were extracted from valid international databases. Additionally, gender inequality was quantified using an extensively used indicator in the literature. Finally, by employing a stepwise multiple linear regression model, we were able to observe gender inequality in a variety of scenarios.

Despite these strengths, our study faces some drawbacks. Firstly, our estimation of DALYs associated with OFCs may be conservative due to the exclusion of related morbidities such as dental or auditory disorders. This underestimation is compounded by variations in DALYs across different subgroups of OFCs, though these subgroup-specific DALYs were not individually assessed. Secondly, the potential underestimation of the OFC burden might vary among countries, with geographical location potentially acting as a confounding variable affecting this estimation. To partially address this issue, we employed macro-level proxy measures like the HDI and SDI to gauge a country’s overall development, which indirectly reflects healthcare systems and potential underestimation of disease burden. However, despite adjusting for these indices in our modeling, the possibility of flawed results remains.

Thirdly, limitations in the 2019 Global Burden of Disease methodology prevented the differentiation between various degrees and types of OFCs, such as unilateral or bilateral clefts, cleft lip, cleft palate, and severity levels, which could significantly impact morbidity, surgical outcomes, and prognosis. Although our study segregated data by gender to examine gender disparities, the inability to differentiate OFC types and severity levels could potentially mask inequalities, particularly affecting females, in global data. Fourthly, incomplete HDI data for multiple countries limited our ability to incorporate comprehensive developmental measures over time, necessitating the use of 2019 data, which may introduce residual confounding effects in the interpretation of our findings. Despite statistical adjustments, the lingering impact of long-term developmental changes remains a consideration in our study’s interpretation.

Lastly, the methodological limitations of the GII in fully capturing societal gender norms hindered a more nuanced analysis of gender-related impacts. Regrettably, comprehensive gender indicators like the Social Institutions and Gender Index (SIGI) and Gender Social Norms Index (GSNI) were unavailable for inclusion.

Future research is better to focus on comparative studies examining OFCs prevalence across diverse ethnicities such as Caucasians, Africans, and other distinct groups. These studies could explore genetic, environmental, and socio-economic factors contributing to varying prevalence rates. Large-scale, standardized studies would deepen our understanding of global OFC distribution, guiding targeted interventions and healthcare strategies tailored to specific populations. These investigations promise insights into complex OFC etiology, advancing prevention and management approaches.

## Conclusion

The burden of orofacial clefts has increased significantly over the previous three decades, despite a decreasing prevalence trend in EMR countries. There is still a strong correlation between the burden and prevalence of OFCs and gender inequality in EMR. Thus, promoting gender equality in public policies and decision-making is critical for the population-level optimization of OFCs’ status. Among the public policies, boosting the resource allocation to the entire population, especially the female population, might be mentioned. In addition, the implementation of projects to reduce the gender gap by international agencies, especially the World Bank, should be considered. Furthermore, the WHO regional office should request from all countries an action plan to reduce the prevalence and burden of the OFCs, which it should then tailor for each country and provide implementation guidance. We also recommend that researchers in this field including the GBD investigators should consider collecting data related to cleft palate disease based on its subtypes and cleft-specific severities.

### Electronic supplementary material

Below is the link to the electronic supplementary material.

Supplementary Material 1: The burden of the orofacial cleft and its correlates in EMR, 1990–2019

## Data Availability

Transcripts related to this study are available to the corresponding author upon a reasonable request. Transcripts will not include any identifiable information if data is requested.

## Abbreviations

OFCs: Orofacial Clefts
EMR: Eastern Mediterranean Region
LMICs: low- and middle-income countries
DALYs: Disability-Adjusted Life Years
GII: Gender Inequality Index
SDI: Socio-demographic Index
GDP: Gross Domestic Product
HDI: Human Developmental Index
SIGI: Social Institutions and Gender Index
GSNI: Gender Social Norms Index
ASR: Age Standardized Rate
EAPC: Estimated Annual Percentage Change
OR: Odds Ratio
GBD: Global Burden of Disease
CI: Confidence Interval
UI: Uncertainty Interval
OLS: Ordinary Least Square
IHME: Institute for Health Metrics and Evaluation
