# Supplementary material for: Gender inequality and burden of orofacial clefts in the Eastern Mediterranean region: findings from global burden of disease study 1990–2019
Source: BMC Pediatr. 2024 Jan 23;24:76. doi: 10.1186/s12887-024-04569-6 (PMC10804627; doi:10.1186/s12887-024-04569-6)
Supplement: Supplementary file 1 — Supplementary Material 1: The burden of the orofacial cleft and its correlates in EMR, 1990–2019 [file 12887_2024_4569_MOESM1_ESM.docx]

Supplementary draft

**Gender inequality is Associated with the Burden of Orofacial Clefts in the Eastern Mediterranean Region: Findings from the Global Burden of Disease Study 1990–2019**

Sara Sadat Nabavizadeh^1,2^, Jennifer J. Mootz^3,4^, Nasser Nadjmi^5,6^, Benjamin B. Massenburg^7^, Kaveh Khoshnood^8^, Ehsan Shojaeefard^1^, Hossein Molavi Vardanjani^1,9^

1. MD-MPH Department, School of Medicine, Shiraz University of Medical Sciences, Shiraz, Iran
2. Otolaryngology Research Center, Shiraz University of Medical Sciences, Shiraz, Iran
3. Department of Psychiatry, Columbia University, 1051 Riverside Drive, New York, NY 10032 USA
4. New York State Psychiatric Institute, 1051 Riverside Drive, Kolb 171, New York, NY 10032 USA
5. Department of Cranio-Maxillofacial Surgery, Antwerp University Hospital - Antwerp, Belgium
6. Department of Maxillofacial Surgery, ZMACK, AZ MONICA Antwerp - Antwerp, Belgium
7. Department of Surgery, Division of Plastic and Reconstructive Surgery, University of Washington, Seattle, Washington, USA
8. School of Public Health, Yale University, 60 College St, New Haven, CT, 06510, USA
9. Research Center for Traditional Medicine and History of Medicine, School of Medicine, Shiraz University of Medical Sciences, Shiraz, Iran

**Contents**

Table. S1. The national prevalence and disability-adjusted life years numbers and age-standardized rates of prevalence and disability-adjusted life years in 1990 and 2019, as well as their temporal trends by estimated annual percentage change from 1990 to 2019.

Fig. S1. Correlation between country-level indicators (human developmental index, and Gross Domestic Product) and orofacial clefts 1990-2019 prevalence and disability-adjusted life years rate, and correlation between socio-demographic index changes and orofacial clefts' 2019 prevalence and disability-adjusted life years.

Table. S1. The national prevalence and disability-adjusted life years numbers and age-standardized rates of prevalence and disability-adjusted life years in 1990 and 2019, as well as their temporal trends by estimated annual percentage change from 1990 to 2019.

| **Location** | **Measure** | **Sex** | **Number 1990** | **ASR 1990** | **Number 2019** | **ASR 2019** | **EAPC** |
| --- | --- | --- | --- | --- | --- | --- | --- |
| **United Arab Emirates** | **Prevalence** | **Male** | 1.37e+03 (1.10e+03 to 1.70e+03) | 113.24 (90.37 to 140.62) | 6.62e+03 (5.23e+03 to 8.40e+03) | 103.69 (82.15 to 130.20) | -0.29 (-0.30 to -0.28) |
|  |  | **Female** | 5.16e+02 (4.09e+02 to 6.47e+02) | 71.00 (55.88 to 88.69) | 1.59e+03 (1.24e+03 to 2.03e+03) | 64.87 (51.28 to 81.94) | -0.26 (-0.28 to -0.24) |
|  |  | **Both** | 1.89e+03 (1.52e+03 to 2.31e+03) | 97.97 (78.91 to 120.07) | 8.21e+03 (6.51e+03 to 1.03e+04) | 89.85 (71.53 to 111.62) | -0.25 (-0.26 to -0.24) |
|  | **DALYs** | **Male** | 9.21e+01 (5.67e+01 to 1.38e+02) | 7.57 (4.67 to 11.18) | 4.14e+02 (2.43e+02 to 6.31e+02) | 6.72 (4.10 to 10.01) | -0.33 (-0.34 to -0.31) |
|  |  | **Female** | 9.93e+01 (5.32e+01 to 1.87e+02) | 10.51 (5.96 to 18.59) | 1.08e+02 (6.60e+01 to 1.63e+02) | 4.76 (2.99 to 7.14) | -2.69 (-2.77 to -2.61) |
|  |  | **Both** | 1.91e+02 (1.18e+02 to 2.91e+02) | 9.33 (5.87 to 13.95) | 5.21e+02 (3.13e+02 to 7.89e+02) | 6.09 (3.83 to 8.89) | -1.37 (-1.41 to -1.33) |
| **Sudan** | **Prevalence** | **Male** | 1.19e+04 (9.57e+03 to 1.47e+04) | 110.75 (89.06 to 137.62) | 2.31e+04 (1.83e+04 to 2.88e+04) | 108.82 (86.92 to 135.93) | 0.00 (-0.01 to 0.01) |
|  |  | **Female** | 9.80e+03 (7.89e+03 to 1.22e+04) | 91.25 (72.73 to 115.11) | 1.79e+04 (1.43e+04 to 2.22e+04) | 86.58 (68.91 to 107.86) | -0.13 (-0.14 to -0.12) |
|  |  | **Both** | 2.17e+04 (1.76e+04 to 2.66e+04) | 101.04 (82.00 to 124.51) | 4.10e+04 (3.32e+04 to 5.09e+04) | 97.91 (78.64 to 121.20) | -0.05 (-0.06 to -0.04) |
|  | **DALYs** | **Male** | 1.16e+03 (6.29e+02 to 2.42e+03) | 8.62 (5.17 to 14.65) | 1.81e+03 (1.09e+03 to 2.83e+03) | 8.00 (4.88 to 12.05) | -0.04 (-0.08 to 0.01) |
|  |  | **Female** | 8.55e+03 (1.45e+03 to 4.34e+04) | 42.89 (9.01 to 208.33) | 2.31e+03 (1.23e+03 to 4.74e+03) | 9.71 (5.41 to 18.76) | -4.80 (-4.87 to -4.74) |
|  |  | **Both** | 9.71e+03 (2.35e+03 to 4.46e+04) | 24.95 (7.80 to 103.05) | 4.12e+03 (2.49e+03 to 7.09e+03) | 8.82 (5.41 to 14.51) | -3.34 (-3.39 to -3.29) |
| **Kuwait** | **Prevalence** | **Male** | 1.21e+03 (9.53e+02 to 1.51e+03) | 119.85 (94.06 to 149.19) | 2.68e+03 (2.11e+03 to 3.34e+03) | 115.27 (91.26 to 143.33) | -0.18 (-0.19 to -0.17) |
|  |  | **Female** | 6.91e+02 (5.45e+02 to 8.52e+02) | 89.58 (70.72 to 111.03) | 1.78e+03 (1.41e+03 to 2.26e+03) | 88.40 (69.83 to 111.63) | -0.06 (-0.07 to -0.05) |
|  |  | **Both** | 1.90e+03 (1.52e+03 to 2.35e+03) | 107.01 (84.99 to 132.21) | 4.46e+03 (3.54e+03 to 5.53e+03) | 102.83 (81.62 to 127.35) | -0.17 (-0.18 to -0.17) |
|  | **DALYs** | **Male** | 7.66e+01 (4.53e+01 to 1.17e+02) | 7.52 (4.50 to 11.39) | 1.65e+02 (1.00e+02 to 2.53e+02) | 7.12 (4.28 to 10.93) | -0.21 (-0.22 to -0.20) |
|  |  | **Female** | 4.59e+01 (2.73e+01 to 7.19e+01) | 5.82 (3.53 to 9.05) | 1.10e+02 (6.85e+01 to 1.69e+02) | 5.48 (3.42 to 8.30) | -0.22 (-0.23 to -0.21) |
|  |  | **Both** | 1.22e+02 (7.32e+01 to 1.87e+02) | 6.81 (4.17 to 10.38) | 2.75e+02 (1.72e+02 to 4.19e+02) | 6.36 (3.98 to 9.64) | -0.26 (-0.27 to -0.25) |
| **Lebanon** | **Prevalence** | **Male** | 1.92e+03 (1.52e+03 to 2.37e+03) | 112.42 (89.22 to 139.76) | 2.77e+03 (2.21e+03 to 3.46e+03) | 108.52 (86.61 to 135.47) | -0.06 (-0.07 to -0.05) |
|  |  | **Female** | 1.54e+03 (1.22e+03 to 1.94e+03) | 90.24 (71.53 to 114.09) | 2.20e+03 (1.74e+03 to 2.76e+03) | 84.02 (66.72 to 105.51) | -0.19 (-0.20 to -0.18) |
|  |  | **Both** | 3.45e+03 (2.78e+03 to 4.23e+03) | 101.30 (81.34 to 124.36) | 4.96e+03 (3.98e+03 to 6.13e+03) | 96.12 (76.95 to 118.69) | -0.13 (-0.13 to -0.12) |
|  | **DALYs** | **Male** | 1.40e+02 (8.84e+01 to 2.10e+02) | 7.72 (4.88 to 11.45) | 1.81e+02 (1.10e+02 to 2.73e+02) | 7.07 (4.29 to 10.76) | -0.20 (-0.23 to -0.18) |
|  |  | **Female** | 3.69e+02 (1.46e+02 to 8.41e+02) | 16.28 (7.38 to 34.66) | 1.62e+02 (1.03e+02 to 2.39e+02) | 6.29 (3.97 to 9.19) | -3.22 (-3.27 to -3.18) |
|  |  | **Both** | 5.09e+02 (2.64e+02 to 9.86e+02) | 11.86 (6.65 to 21.08) | 3.43e+02 (2.18e+02 to 5.01e+02) | 6.66 (4.24 to 9.75) | -1.91 (-1.94 to -1.88) |
| **Yemen** | **Prevalence** | **Male** | 8.04e+03 (6.47e+03 to 9.93e+03) | 108.28 (87.17 to 135.16) | 1.77e+04 (1.41e+04 to 2.20e+04) | 107.68 (86.03 to 134.53) | 0.01 (-0.00 to 0.03) |
|  |  | **Female** | 6.57e+03 (5.24e+03 to 8.15e+03) | 88.70 (70.35 to 110.54) | 1.35e+04 (1.06e+04 to 1.66e+04) | 84.01 (66.35 to 103.49) | -0.22 (-0.23 to -0.21) |
|  |  | **Both** | 1.46e+04 (1.19e+04 to 1.78e+04) | 98.49 (80.18 to 120.67) | 3.11e+04 (2.49e+04 to 3.80e+04) | 95.91 (76.40 to 117.57) | -0.09 (-0.10 to -0.08) |
|  | **DALYs** | **Male** | 8.73e+02 (4.20e+02 to 1.97e+03) | 8.98 (5.05 to 16.18) | 1.50e+03 (9.40e+02 to 2.61e+03) | 8.38 (5.32 to 13.61) | -0.24 (-0.26 to -0.23) |
|  |  | **Female** | 6.00e+03 (1.10e+03 to 3.11e+04) | 41.97 (9.49 to 207.31) | 2.13e+03 (9.97e+02 to 5.04e+03) | 11.07 (5.60 to 24.49) | -5.31 (-5.50 to -5.12) |
|  |  | **Both** | 6.87e+03 (1.77e+03 to 3.21e+04) | 25.05 (7.99 to 107.83) | 3.62e+03 (2.07e+03 to 6.95e+03) | 9.68 (5.82 to 17.19) | -3.79 (-3.94 to -3.64) |
| **Libya** | **Prevalence** | **Male** | 2.54e+03 (2.02e+03 to 3.17e+03) | 110.63 (87.76 to 138.47) | 3.64e+03 (2.88e+03 to 4.51e+03) | 106.39 (84.61 to 131.96) | -0.12 (-0.12 to -0.11) |
|  |  | **Female** | 1.91e+03 (1.54e+03 to 2.37e+03) | 87.43 (69.28 to 109.34) | 2.64e+03 (2.08e+03 to 3.32e+03) | 82.82 (65.56 to 103.91) | -0.16 (-0.17 to -0.15) |
|  |  | **Both** | 4.46e+03 (3.59e+03 to 5.49e+03) | 99.86 (80.69 to 122.82) | 6.27e+03 (5.01e+03 to 7.77e+03) | 94.96 (76.26 to 117.27) | -0.14 (-0.15 to -0.14) |
|  | **DALYs** | **Male** | 1.83e+02 (1.13e+02 to 2.73e+02) | 7.50 (4.63 to 11.20) | 2.32e+02 (1.41e+02 to 3.48e+02) | 6.93 (4.27 to 10.36) | -0.23 (-0.24 to -0.22) |
|  |  | **Female** | 5.11e+02 (2.21e+02 to 1.61e+03) | 16.40 (7.78 to 46.77) | 1.85e+02 (1.17e+02 to 2.70e+02) | 6.30 (4.07 to 9.06) | -3.18 (-3.25 to -3.10) |
|  |  | **Both** | 6.94e+02 (3.69e+02 to 1.78e+03) | 11.93 (6.87 to 26.41) | 4.17e+02 (2.63e+02 to 6.12e+02) | 6.62 (4.28 to 9.58) | -1.91 (-1.96 to -1.86) |
| **Morocco** | **Prevalence** | **Male** | 1.34e+04 (1.06e+04 to 1.66e+04) | 103.31 (82.11 to 127.72) | 1.85e+04 (1.45e+04 to 2.31e+04) | 102.95 (80.93 to 128.15) | -0.01 (-0.02 to 0.01) |
|  |  | **Female** | 1.10e+04 (8.75e+03 to 1.38e+04) | 83.01 (65.77 to 104.58) | 1.42e+04 (1.12e+04 to 1.79e+04) | 80.16 (63.58 to 101.03) | -0.16 (-0.17 to -0.15) |
|  |  | **Both** | 2.44e+04 (1.98e+04 to 2.99e+04) | 93.06 (75.54 to 114.67) | 3.26e+04 (2.61e+04 to 4.10e+04) | 91.62 (73.13 to 114.65) | -0.07 (-0.08 to -0.06) |
|  | **DALYs** | **Male** | 1.01e+03 (6.15e+02 to 1.64e+03) | 7.32 (4.51 to 11.63) | 1.22e+03 (7.62e+02 to 1.84e+03) | 6.85 (4.31 to 10.26) | -0.20 (-0.21 to -0.18) |
|  |  | **Female** | 2.74e+03 (1.05e+03 to 8.21e+03) | 16.29 (6.83 to 46.25) | 1.07e+03 (6.56e+02 to 1.66e+03) | 6.36 (3.86 to 10.09) | -3.64 (-3.74 to -3.54) |
|  |  | **Both** | 3.75e+03 (1.82e+03 to 9.36e+03) | 11.70 (6.09 to 26.40) | 2.29e+03 (1.46e+03 to 3.40e+03) | 6.60 (4.21 to 9.81) | -2.20 (-2.26 to -2.14) |
| **Afghanistan** | **Prevalence** | **Male** | 6.17e+03 (4.96e+03 to 7.63e+03) | 104.24 (83.78 to 129.16) | 2.02e+04 (1.62e+04 to 2.53e+04) | 98.81 (79.26 to 123.10) | -0.22 (-0.23 to -0.21) |
|  |  | **Female** | 5.37e+03 (4.31e+03 to 6.63e+03) | 86.67 (69.07 to 107.92) | 1.58e+04 (1.26e+04 to 1.95e+04) | 80.18 (64.23 to 99.31) | -0.35 (-0.36 to -0.34) |
|  |  | **Both** | 1.15e+04 (9.37e+03 to 1.40e+04) | 95.15 (77.08 to 116.70) | 3.60e+04 (2.90e+04 to 4.40e+04) | 89.62 (72.00 to 110.55) | -0.27 (-0.28 to -0.26) |
|  | **DALYs** | **Male** | 7.87e+02 (3.75e+02 to 1.69e+03) | 9.66 (5.43 to 17.35) | 2.44e+03 (1.27e+03 to 5.03e+03) | 9.32 (5.40 to 16.81) | -0.03 (-0.17 to 0.11) |
|  |  | **Female** | 6.64e+03 (1.21e+03 to 3.33e+04) | 60.15 (12.38 to 291.83) | 4.69e+03 (1.81e+03 to 1.48e+04) | 15.79 (6.87 to 45.10) | -5.09 (-5.27 to -4.90) |
|  |  | **Both** | 7.42e+03 (1.77e+03 to 3.39e+04) | 34.10 (9.66 to 145.75) | 7.13e+03 (3.40e+03 to 1.73e+04) | 12.44 (6.68 to 26.92) | -3.83 (-3.98 to -3.68) |
| **Palestine** | **Prevalence** | **Male** | 1.73e+03 (1.39e+03 to 2.13e+03) | 155.62 (124.95 to 193.56) | 3.88e+03 (3.10e+03 to 4.81e+03) | 150.83 (120.26 to 186.93) | -0.09 (-0.12 to -0.07) |
|  |  | **Female** | 1.49e+03 (1.20e+03 to 1.84e+03) | 135.81 (107.67 to 168.58) | 3.16e+03 (2.54e+03 to 3.92e+03) | 127.75 (102.64 to 158.54) | -0.24 (-0.26 to -0.22) |
|  |  | **Both** | 3.22e+03 (2.59e+03 to 3.95e+03) | 145.53 (116.67 to 179.25) | 7.05e+03 (5.72e+03 to 8.65e+03) | 139.41 (113.56 to 171.37) | -0.16 (-0.18 to -0.14) |
|  | **DALYs** | **Male** | 2.78e+02 (1.25e+02 to 6.19e+02) | 16.94 (9.13 to 32.19) | 2.49e+02 (1.52e+02 to 3.75e+02) | 9.53 (5.86 to 14.44) | -2.18 (-2.23 to -2.13) |
|  |  | **Female** | 3.69e+02 (1.67e+02 to 1.01e+03) | 20.89 (10.44 to 50.26) | 2.26e+02 (1.48e+02 to 3.29e+02) | 8.85 (5.73 to 12.83) | -3.15 (-3.21 to -3.10) |
|  |  | **Both** | 6.47e+02 (3.30e+02 to 1.45e+03) | 18.83 (10.88 to 36.59) | 4.75e+02 (3.02e+02 to 6.94e+02) | 9.19 (5.81 to 13.38) | -2.67 (-2.72 to -2.63) |
| **Bahrain** | **Prevalence** | **Male** | 3.21e+02 (2.56e+02 to 3.97e+02) | 108.19 (86.21 to 133.73) | 9.04e+02 (7.06e+02 to 1.12e+03) | 104.36 (81.73 to 129.12) | -0.17 (-0.19 to -0.15) |
|  |  | **Female** | 2.05e+02 (1.64e+02 to 2.57e+02) | 90.15 (71.56 to 112.08) | 4.32e+02 (3.38e+02 to 5.45e+02) | 81.40 (64.27 to 102.20) | -0.45 (-0.47 to -0.43) |
|  |  | **Both** | 5.25e+02 (4.30e+02 to 6.39e+02) | 100.72 (82.55 to 122.62) | 1.34e+03 (1.07e+03 to 1.64e+03) | 94.77 (76.07 to 116.08) | -0.26 (-0.28 to -0.24) |
|  | **DALYs** | **Male** | 2.11e+01 (1.30e+01 to 3.15e+01) | 7.00 (4.30 to 10.44) | 5.63e+01 (3.47e+01 to 8.66e+01) | 6.61 (4.10 to 10.00) | -0.23 (-0.25 to -0.21) |
|  |  | **Female** | 5.18e+01 (2.38e+01 to 1.03e+02) | 17.72 (8.72 to 33.98) | 3.10e+01 (2.00e+01 to 4.63e+01) | 6.44 (4.21 to 9.39) | -3.87 (-4.02 to -3.72) |
|  |  | **Both** | 7.28e+01 (4.10e+01 to 1.24e+02) | 12.33 (7.22 to 20.32) | 8.73e+01 (5.51e+01 to 1.30e+02) | 6.63 (4.37 to 9.72) | -2.36 (-2.46 to -2.25) |
| **Iran (Islamic Republic of)** | **Prevalence** | **Male** | 2.70e+04 (2.18e+04 to 3.29e+04) | 86.97 (70.31 to 106.62) | 3.57e+04 (2.89e+04 to 4.42e+04) | 84.33 (68.22 to 104.00) | -0.12 (-0.14 to -0.11) |
|  |  | **Female** | 2.09e+04 (1.70e+04 to 2.58e+04) | 69.08 (55.55 to 85.91) | 2.73e+04 (2.19e+04 to 3.40e+04) | 66.81 (53.54 to 82.71) | -0.35 (-0.39 to -0.31) |
|  |  | **Both** | 4.79e+04 (3.88e+04 to 5.83e+04) | 78.21 (63.22 to 95.54) | 6.31e+04 (5.12e+04 to 7.74e+04) | 75.72 (61.57 to 92.91) | -0.22 (-0.24 to -0.21) |
|  | **DALYs** | **Male** | 2.19e+03 (1.41e+03 to 3.23e+03) | 6.50 (4.24 to 9.49) | 2.38e+03 (1.53e+03 to 3.50e+03) | 5.71 (3.75 to 8.40) | -0.41 (-0.44 to -0.38) |
|  |  | **Female** | 7.68e+03 (3.24e+03 to 2.66e+04) | 19.40 (8.73 to 64.37) | 2.21e+03 (1.51e+03 to 3.07e+03) | 5.78 (3.96 to 7.95) | -4.43 (-4.53 to -4.33) |
|  |  | **Both** | 9.87e+03 (5.02e+03 to 2.89e+04) | 12.80 (6.93 to 34.79) | 4.59e+03 (3.07e+03 to 6.60e+03) | 5.74 (3.86 to 8.13) | -2.89 (-2.95 to -2.83) |
| **Pakistan** | **Prevalence** | **Male** | 6.60e+04 (5.35e+04 to 8.18e+04) | 105.27 (85.23 to 130.20) | 1.28e+05 (1.03e+05 to 1.59e+05) | 108.00 (86.42 to 134.52) | -0.04 (-0.06 to -0.02) |
|  |  | **Female** | 5.35e+04 (4.30e+04 to 6.64e+04) | 93.70 (75.31 to 116.61) | 1.10e+05 (8.86e+04 to 1.37e+05) | 98.02 (78.23 to 122.83) | 0.09 (0.07 to 0.11) |
|  |  | **Both** | 1.19e+05 (9.62e+04 to 1.48e+05) | 99.79 (80.54 to 123.89) | 2.38e+05 (1.91e+05 to 2.94e+05) | 103.13 (82.48 to 128.41) | 0.01 (-0.01 to 0.03) |
|  | **DALYs** | **Male** | 2.50e+04 (6.71e+03 to 6.82e+04) | 24.24 (8.46 to 61.40) | 1.71e+04 (8.23e+03 to 3.35e+04) | 12.23 (6.24 to 22.76) | -2.46 (-2.51 to -2.42) |
|  |  | **Female** | 1.26e+04 (4.71e+03 to 3.37e+04) | 14.12 (6.56 to 33.42) | 1.32e+04 (7.53e+03 to 2.30e+04) | 10.17 (6.04 to 16.79) | -1.13 (-1.17 to -1.08) |
|  |  | **Both** | 3.76e+04 (1.27e+04 to 9.08e+04) | 19.35 (7.92 to 42.08) | 3.03e+04 (1.69e+04 to 5.33e+04) | 11.23 (6.73 to 18.75) | -1.94 (-1.99 to -1.89) |
| **Egypt** | **Prevalence** | **Male** | 3.17e+04 (2.53e+04 to 3.94e+04) | 107.60 (85.54 to 133.76) | 5.50e+04 (4.35e+04 to 6.79e+04) | 106.28 (84.09 to 131.25) | -0.04 (-0.06 to -0.03) |
|  |  | **Female** | 2.44e+04 (1.95e+04 to 3.05e+04) | 84.93 (67.45 to 106.95) | 4.03e+04 (3.18e+04 to 5.04e+04) | 83.44 (65.67 to 104.67) | -0.04 (-0.06 to -0.02) |
|  |  | **Both** | 5.61e+04 (4.56e+04 to 6.88e+04) | 96.49 (78.04 to 118.54) | 9.53e+04 (7.65e+04 to 1.18e+05) | 95.37 (76.38 to 117.92) | -0.03 (-0.04 to -0.02) |
|  | **DALYs** | **Male** | 2.34e+03 (1.41e+03 to 3.60e+03) | 7.44 (4.58 to 11.27) | 3.64e+03 (2.18e+03 to 5.49e+03) | 6.99 (4.20 to 10.48) | -0.16 (-0.19 to -0.13) |
|  |  | **Female** | 6.24e+03 (2.81e+03 to 1.60e+04) | 15.98 (7.82 to 38.33) | 3.08e+03 (1.88e+03 to 4.68e+03) | 6.30 (3.89 to 9.60) | -3.25 (-3.29 to -3.22) |
|  |  | **Both** | 8.57e+03 (4.59e+03 to 1.82e+04) | 11.60 (6.63 to 22.15) | 6.72e+03 (4.26e+03 to 1.00e+04) | 6.67 (4.24 to 9.93) | -1.92 (-1.94 to -1.90) |
| **Qatar** | **Prevalence** | **Male** | 4.57e+02 (3.62e+02 to 5.72e+02) | 154.81 (122.68 to 193.42) | 2.95e+03 (2.32e+03 to 3.67e+03) | 143.04 (112.70 to 176.90) | -0.32 (-0.33 to -0.31) |
|  |  | **Female** | 1.56e+02 (1.25e+02 to 1.95e+02) | 101.72 (80.67 to 127.88) | 7.41e+02 (5.87e+02 to 9.29e+02) | 102.07 (80.87 to 127.83) | -0.03 (-0.04 to -0.02) |
|  |  | **Both** | 6.13e+02 (4.89e+02 to 7.62e+02) | 135.37 (108.03 to 168.71) | 3.69e+03 (2.93e+03 to 4.56e+03) | 130.30 (103.80 to 160.32) | -0.15 (-0.15 to -0.14) |
|  | **DALYs** | **Male** | 3.39e+01 (2.12e+01 to 5.14e+01) | 11.68 (7.47 to 17.69) | 1.86e+02 (1.12e+02 to 2.87e+02) | 9.26 (5.82 to 13.61) | -0.86 (-0.89 to -0.84) |
|  |  | **Female** | 1.03e+01 (6.44e+00 to 1.53e+01) | 6.46 (3.93 to 9.52) | 4.65e+01 (2.81e+01 to 7.03e+01) | 6.36 (3.89 to 9.66) | -0.09 (-0.10 to -0.08) |
|  |  | **Both** | 4.42e+01 (2.81e+01 to 6.60e+01) | 9.54 (6.16 to 14.24) | 2.33e+02 (1.42e+02 to 3.53e+02) | 8.30 (5.18 to 12.29) | -0.50 (-0.52 to -0.49) |
| **Oman** | **Prevalence** | **Male** | 1.47e+03 (1.16e+03 to 1.84e+03) | 125.75 (99.08 to 158.20) | 3.49e+03 (2.77e+03 to 4.30e+03) | 120.44 (95.74 to 147.42) | -0.08 (-0.09 to -0.07) |
|  |  | **Female** | 8.61e+02 (6.84e+02 to 1.08e+03) | 97.31 (76.79 to 123.43) | 1.54e+03 (1.22e+03 to 1.95e+03) | 93.34 (73.99 to 117.79) | -0.08 (-0.10 to -0.05) |
|  |  | **Both** | 2.33e+03 (1.86e+03 to 2.87e+03) | 114.10 (91.04 to 141.55) | 5.04e+03 (4.03e+03 to 6.16e+03) | 109.70 (87.71 to 133.95) | -0.06 (-0.08 to -0.04) |
|  | **DALYs** | **Male** | 1.03e+02 (6.27e+01 to 1.54e+02) | 8.47 (5.25 to 12.52) | 2.23e+02 (1.34e+02 to 3.46e+02) | 7.74 (4.61 to 11.48) | -0.18 (-0.21 to -0.16) |
|  |  | **Female** | 1.83e+02 (7.64e+01 to 5.64e+02) | 14.31 (6.76 to 38.63) | 1.08e+02 (6.83e+01 to 1.61e+02) | 6.42 (4.05 to 9.54) | -2.36 (-2.55 to -2.17) |
|  |  | **Both** | 2.86e+02 (1.53e+02 to 6.75e+02) | 11.47 (6.69 to 23.31) | 3.31e+02 (2.07e+02 to 4.89e+02) | 7.26 (4.63 to 10.62) | -1.26 (-1.38 to -1.15) |
| **Saudi Arabia** | **Prevalence** | **Male** | 9.40e+03 (7.48e+03 to 1.17e+04) | 101.78 (81.02 to 127.81) | 2.02e+04 (1.60e+04 to 2.52e+04) | 99.23 (78.74 to 123.06) | -0.11 (-0.12 to -0.10) |
|  |  | **Female** | 6.59e+03 (5.25e+03 to 8.27e+03) | 86.41 (68.56 to 110.02) | 1.24e+04 (9.85e+03 to 1.55e+04) | 84.67 (66.98 to 105.06) | -0.04 (-0.06 to -0.03) |
|  |  | **Both** | 1.60e+04 (1.30e+04 to 1.98e+04) | 95.32 (77.01 to 118.63) | 3.26e+04 (2.61e+04 to 4.05e+04) | 93.02 (74.03 to 114.81) | -0.09 (-0.10 to -0.08) |
|  | **DALYs** | **Male** | 6.88e+02 (4.10e+02 to 1.08e+03) | 7.06 (4.32 to 10.75) | 1.28e+03 (7.98e+02 to 1.96e+03) | 6.39 (3.93 to 9.60) | -0.34 (-0.36 to -0.32) |
|  |  | **Female** | 1.61e+03 (5.90e+02 to 5.37e+03) | 14.80 (6.27 to 44.94) | 8.27e+02 (4.97e+02 to 1.27e+03) | 5.78 (3.56 to 8.71) | -3.44 (-3.54 to -3.34) |
|  |  | **Both** | 2.29e+03 (1.08e+03 to 6.23e+03) | 10.94 (5.66 to 26.50) | 2.11e+03 (1.29e+03 to 3.17e+03) | 6.15 (3.83 to 9.15) | -2.06 (-2.12 to -2.00) |
| **Djibouti** | **Prevalence** | **Male** | 1.53e+02 (1.23e+02 to 1.91e+02) | 54.08 (42.97 to 68.18) | 3.21e+02 (2.55e+02 to 4.00e+02) | 48.89 (38.76 to 61.11) | -0.44 (-0.45 to -0.43) |
|  |  | **Female** | 1.21e+02 (9.66e+01 to 1.51e+02) | 48.68 (38.50 to 60.91) | 2.56e+02 (2.05e+02 to 3.22e+02) | 43.97 (34.81 to 56.02) | -0.40 (-0.41 to -0.40) |
|  |  | **Both** | 2.74e+02 (2.22e+02 to 3.38e+02) | 51.57 (41.20 to 64.44) | 5.77e+02 (4.62e+02 to 7.20e+02) | 46.59 (37.15 to 58.26) | -0.42 (-0.43 to -0.41) |
|  | **DALYs** | **Male** | 3.82e+01 (1.14e+01 to 1.50e+02) | 8.72 (3.24 to 29.62) | 5.02e+01 (2.30e+01 to 1.37e+02) | 6.57 (3.22 to 17.29) | -0.88 (-0.99 to -0.76) |
|  |  | **Female** | 6.92e+01 (1.48e+01 to 3.39e+02) | 15.38 (4.18 to 69.83) | 6.36e+01 (2.32e+01 to 2.26e+02) | 8.66 (3.49 to 28.80) | -1.93 (-2.10 to -1.76) |
|  |  | **Both** | 1.07e+02 (2.93e+01 to 3.71e+02) | 11.96 (4.10 to 38.07) | 1.14e+02 (4.94e+01 to 2.82e+02) | 7.59 (3.53 to 17.68) | -1.50 (-1.64 to -1.35) |
| **Syrian Arab Republic** | **Prevalence** | **Male** | 7.29e+03 (5.74e+03 to 9.05e+03) | 105.21 (82.45 to 130.50) | 7.45e+03 (5.94e+03 to 9.28e+03) | 106.13 (84.77 to 131.91) | 0.08 (0.07 to 0.09) |
|  |  | **Female** | 5.40e+03 (4.27e+03 to 6.75e+03) | 80.21 (63.36 to 100.71) | 6.15e+03 (4.84e+03 to 7.68e+03) | 83.87 (66.30 to 104.33) | 0.29 (0.27 to 0.31) |
|  |  | **Both** | 1.27e+04 (1.01e+04 to 1.56e+04) | 93.03 (74.36 to 114.61) | 1.36e+04 (1.09e+04 to 1.67e+04) | 94.65 (75.71 to 116.18) | 0.15 (0.13 to 0.16) |
|  | **DALYs** | **Male** | 6.32e+02 (3.76e+02 to 1.02e+03) | 7.93 (4.93 to 12.28) | 5.00e+02 (3.13e+02 to 7.50e+02) | 7.22 (4.53 to 10.58) | -0.18 (-0.20 to -0.16) |
|  |  | **Female** | 6.35e+02 (3.70e+02 to 1.13e+03) | 7.48 (4.58 to 11.93) | 4.05e+02 (2.48e+02 to 5.98e+02) | 5.66 (3.51 to 8.27) | -0.70 (-0.74 to -0.65) |
|  |  | **Both** | 1.27e+03 (8.05e+02 to 1.93e+03) | 7.71 (5.01 to 11.41) | 9.05e+02 (5.70e+02 to 1.31e+03) | 6.42 (4.10 to 9.27) | -0.43 (-0.46 to -0.40) |
| **Somalia** | **Prevalence** | **Male** | 2.15e+03 (1.74e+03 to 2.66e+03) | 54.03 (43.84 to 67.11) | 5.79e+03 (4.61e+03 to 7.13e+03) | 52.20 (41.12 to 64.26) | -0.10 (-0.12 to -0.09) |
|  |  | **Female** | 1.77e+03 (1.41e+03 to 2.23e+03) | 47.78 (37.76 to 60.57) | 4.90e+03 (3.84e+03 to 6.02e+03) | 45.99 (36.33 to 56.85) | -0.12 (-0.13 to -0.11) |
|  |  | **Both** | 3.92e+03 (3.18e+03 to 4.82e+03) | 50.94 (41.11 to 63.17) | 1.07e+04 (8.56e+03 to 1.30e+04) | 49.06 (39.05 to 60.50) | -0.11 (-0.12 to -0.11) |
|  | **DALYs** | **Male** | 1.17e+03 (1.94e+02 to 5.52e+03) | 17.13 (3.79 to 74.79) | 3.35e+03 (5.91e+02 to 1.62e+04) | 17.53 (3.98 to 78.12) | 0.98 (0.86 to 1.11) |
|  |  | **Female** | 1.46e+03 (2.02e+02 to 7.48e+03) | 22.05 (4.03 to 107.76) | 3.42e+03 (5.61e+02 to 1.79e+04) | 18.50 (3.83 to 90.38) | 0.31 (0.17 to 0.44) |
|  |  | **Both** | 2.63e+03 (4.59e+02 to 9.60e+03) | 19.51 (4.29 to 67.75) | 6.77e+03 (1.35e+03 to 2.86e+04) | 18.00 (4.51 to 71.60) | 0.63 (0.50 to 0.76) |
| **Iraq** | **Prevalence** | **Male** | 1.07e+04 (8.52e+03 to 1.33e+04) | 112.25 (88.76 to 139.91) | 2.37e+04 (1.90e+04 to 2.94e+04) | 108.50 (86.94 to 134.94) | -0.11 (-0.13 to -0.10) |
|  |  | **Female** | 8.48e+03 (6.74e+03 to 1.06e+04) | 89.42 (70.67 to 112.25) | 1.72e+04 (1.35e+04 to 2.15e+04) | 82.87 (65.15 to 103.57) | -0.31 (-0.32 to -0.30) |
|  |  | **Both** | 1.92e+04 (1.54e+04 to 2.34e+04) | 101.00 (80.73 to 123.55) | 4.09e+04 (3.29e+04 to 5.05e+04) | 95.93 (77.08 to 118.52) | -0.20 (-0.21 to -0.19) |
|  | **DALYs** | **Male** | 1.11e+03 (6.12e+02 to 1.99e+03) | 9.51 (5.74 to 15.54) | 1.61e+03 (1.01e+03 to 2.43e+03) | 7.26 (4.56 to 10.81) | -0.84 (-0.88 to -0.80) |
|  |  | **Female** | 4.03e+03 (1.71e+03 to 1.23e+04) | 26.93 (12.31 to 77.45) | 1.51e+03 (9.80e+02 to 2.28e+03) | 7.08 (4.61 to 10.57) | -4.57 (-4.66 to -4.49) |
|  |  | **Both** | 5.14e+03 (2.61e+03 to 1.32e+04) | 17.99 (9.81 to 41.38) | 3.12e+03 (2.07e+03 to 4.54e+03) | 7.17 (4.74 to 10.37) | -3.11 (-3.19 to -3.04) |
| **Jordan** | **Prevalence** | **Male** | 2.46e+03 (1.97e+03 to 3.09e+03) | 117.79 (93.25 to 150.08) | 6.84e+03 (5.44e+03 to 8.60e+03) | 109.63 (87.34 to 138.03) | -0.34 (-0.35 to -0.32) |
|  |  | **Female** | 1.73e+03 (1.40e+03 to 2.15e+03) | 89.06 (71.00 to 111.80) | 4.61e+03 (3.66e+03 to 5.75e+03) | 84.14 (66.80 to 105.02) | -0.28 (-0.30 to -0.26) |
|  |  | **Both** | 4.19e+03 (3.44e+03 to 5.19e+03) | 104.06 (84.30 to 130.07) | 1.14e+04 (9.15e+03 to 1.43e+04) | 97.73 (78.13 to 121.71) | -0.30 (-0.32 to -0.28) |
|  | **DALYs** | **Male** | 5.89e+02 (2.82e+02 to 1.16e+03) | 20.32 (10.60 to 37.06) | 6.63e+02 (4.24e+02 to 9.65e+02) | 10.70 (6.79 to 15.41) | -2.39 (-2.48 to -2.30) |
|  |  | **Female** | 7.57e+02 (3.74e+02 to 1.57e+03) | 25.95 (13.52 to 51.60) | 6.09e+02 (3.97e+02 to 9.15e+02) | 10.78 (7.07 to 16.08) | -3.57 (-3.77 to -3.37) |
|  |  | **Both** | 1.35e+03 (8.29e+02 to 2.46e+03) | 23.08 (14.73 to 40.14) | 1.27e+03 (8.76e+02 to 1.74e+03) | 10.77 (7.45 to 14.76) | -2.99 (-3.13 to -2.85) |
| **Tunisia** | **Prevalence** | **Male** | 4.38e+03 (3.50e+03 to 5.41e+03) | 100.07 (79.78 to 123.76) | 5.59e+03 (4.46e+03 to 6.96e+03) | 98.12 (78.24 to 122.00) | -0.03 (-0.04 to -0.02) |
|  |  | **Female** | 3.54e+03 (2.83e+03 to 4.44e+03) | 81.88 (65.28 to 102.70) | 4.41e+03 (3.47e+03 to 5.61e+03) | 77.66 (61.64 to 98.73) | -0.14 (-0.15 to -0.13) |
|  |  | **Both** | 7.92e+03 (6.43e+03 to 9.68e+03) | 91.05 (73.69 to 111.25) | 1.00e+04 (8.03e+03 to 1.24e+04) | 87.87 (70.89 to 108.37) | -0.08 (-0.09 to -0.07) |
|  | **DALYs** | **Male** | 3.67e+02 (2.19e+02 to 5.63e+02) | 7.79 (4.73 to 11.67) | 3.72e+02 (2.31e+02 to 5.52e+02) | 6.71 (4.24 to 9.88) | -0.39 (-0.41 to -0.36) |
|  |  | **Female** | 1.28e+03 (5.52e+02 to 3.52e+03) | 24.39 (10.91 to 65.65) | 3.31e+02 (2.07e+02 to 4.85e+02) | 6.34 (4.05 to 9.23) | -4.59 (-4.67 to -4.52) |
|  |  | **Both** | 1.65e+03 (8.49e+02 to 4.01e+03) | 15.80 (8.49 to 36.85) | 7.04e+02 (4.43e+02 to 1.03e+03) | 6.50 (4.10 to 9.43) | -3.00 (-3.05 to -2.94) |

Table S2. Correlation between country-level indicators and orofacial clefts 1990-2019 prevalence and disability-adjusted life years rate stratified by human developmental index (HD)

|  |  |  | **GII** | **HDI** | **Δ SDI** | **GII-HDI  Interaction** | **Year** | **R-squared** |  |
| --- | --- | --- | --- | --- | --- | --- | --- | --- | --- |
|  |  |  |  |  |  |  |  |  |  |
| **Low HDI** | **Prevalence** | **Adjusted β** | -2.18 | -0.98 | 0.413 | 1.577 | -0.18 | 0.82 |  |
|  |  | **Std Error** | 67.79 | 103.26 | 20.21 | 135.69 | 0.177 |  |  |
|  |  | **P-value** | <0.001 | 0.004 | 0.002 | 0.001 | <0.001 |  |  |
|  | **DALYs** | **Adjusted β** | -2.55 | -2.21 | -0.26 | 2.21 | -0.22 | 0.79 |  |
|  |  | **Std Error** | 17.23 | 2625 | 5.13 | 34.51 | 0.04 |  |  |
|  |  | **P-value** | <0.001 | <0.001 | 0.001 | <0.001 | 0.001 |  |  |
| **Medium HDI** | **Prevalence** | **Adjusted β** | -0.06 | 0.18 | 0.19 | 0.92 | 0.03 | 0.85 |  |
|  |  | **Std Error** | 105.64 | 79.27 | 19.56 | 150.71 | 0.11 |  |  |
|  |  | **P-value** | 0.967 | 0.405 | <0.001 | 0.519 | 0.409 |  |  |
|  | **DALYs** | **Adjusted β** | 3.47 | 0.93 | -0.31 | -2.93 | -0.69 | 0.69 |  |
|  |  | **Std Error** | 24.21 | 18.16 | 4.48 | 34.53 | 0.02 |  |  |
|  |  | **P-value** | 0.101 | 0.004 | <0.001 | 0.157 | <0.001 |  |  |
| **High HDI** | **Prevalence** | **Adjusted β** | -8.75 | -0.65 | -0.08 | 8.51 | -0.21 | 0.29 |  |
|  |  | **Std Error** | 195.83 | 93.94 | 25.68 | 240.94 | 0.21 |  |  |
|  |  | **P-value** | <0.001 | 0.008 | 0.338 | <0.001 | 0.003 |  |  |
|  | **DALYs** | **Adjusted β** | -5.36 | -0.57 | 0.03 | 5.08 | -0.66 | 0.47 |  |
|  |  | **Std Error** | 11.91 | 5.71 | 1.56 | 14.65 | 0.01 |  |  |
|  |  | **P-value** | 0.001 | 0.007 | 0.599 | 0.001 | <0.001 |  |  |

*Abbreviations: DALYs: Disability-adjusted life years; Human Developmental Index (HDI), Socio-Demographic Index changes: (ΔSDI 2019-1990); Gender inequality index; GII; s


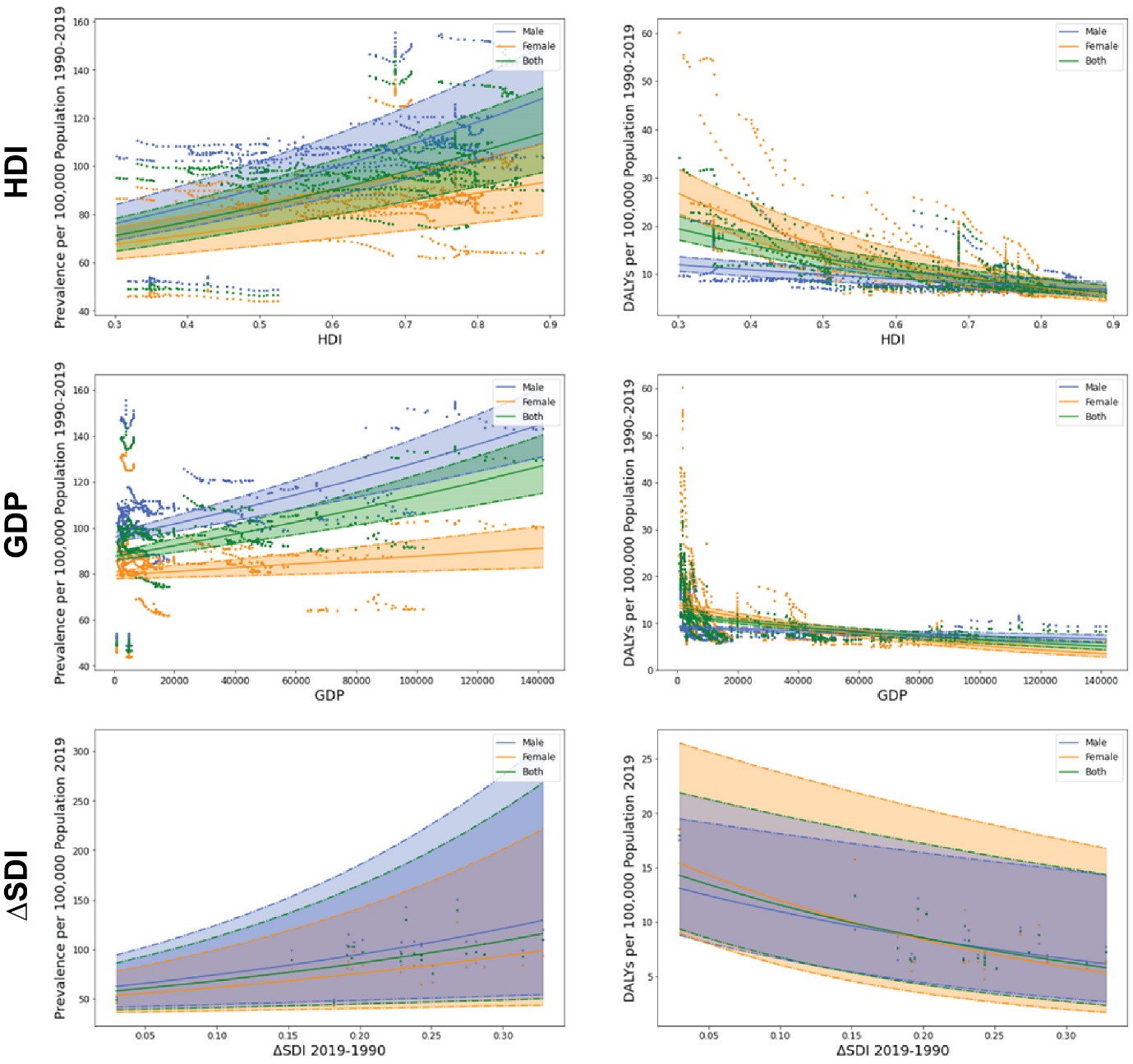


Fig. S1. Correlation between country-level indicators (human developmental index, and Gross Domestic Product) and orofacial clefts 1990-2019 prevalence and disability-adjusted life years rate, and correlation between socio-demographic index changes and orofacial clefts' 2019 prevalence and disability-adjusted life years.
